# Risk Factors of Low Bone Mineral Density in Newly Diagnosed Pediatric Inflammatory Bowel Disease

**DOI:** 10.3390/nu15245048

**Published:** 2023-12-08

**Authors:** Moon Bae Ahn, In Hyuk Yoo

**Affiliations:** Department of Pediatrics, College of Medicine, The Catholic University of Korea, Seoul 06591, Republic of Korea; mbahn@catholic.ac.kr

**Keywords:** inflammatory bowel diseases, bone mineral density, body mass index, osteoporosis

## Abstract

Inflammatory bowel disease (IBD) is a chronic inflammatory disorder of the gastrointestinal tract with an increasing worldwide incidence. IBD is frequently diagnosed during childhood in the adolescent period of ongoing growth and development, and it can affect patients’ linear growth, puberty, nutrition, and bone health. Therefore, its treatment and monitoring are critical to prevent secondary outcomes. However, few studies have highlighted the association between pediatric IBD and skeletal outcomes in Asian populations. We aimed to identify the prevalence and risk factors for low bone mineral density (BMD) in Korean children and adolescents with newly diagnosed IBD. Patients aged 10–18 years diagnosed with either Crohn’s disease (CD) or ulcerative colitis (UC) who underwent lumbar spine bone mineral density (LSBMD) and femoral bone mineral density (FBMD) analyses via dual-energy X-ray absorptiometry at the time of IBD diagnosis were included. Low BMD was considered when the age- and sex-matched BMD *Z*-score was <−1.0. The LSBMD and FBMD *Z*-scores were correlated with clinical parameters, including general characteristics, anthropometry, and IBD-associated laboratory markers. Regression analyses were performed to identify the risk factors for low BMD. Although the general characteristics between CD (*n* = 42) and UC (*n* = 9) groups did not differ, the mean *Z*-scores of LSBMD and FBMD of the 51 subjects were −0.11 ± 1.24 and −0.58 ± 1.38, respectively. Furthermore, 7.8% and 18% of the study subjects had LSBMD and FBMD *Z*-scores < −2.0, whereas more than 50% had scores of 0–−1.0. Among the clinical factors, body mass index (BMI) *Z*-score, duration of clinical manifestations, and serum alanine aminotransferase and selenium levels were associated with LSBMD *Z*-scores in the final multivariate regression analyses. Odds ratios of BMI < −2.0 standard deviation for low LSBMD and FBMD *Z*-scores were 31.97 and 41.45, respectively. A BMI *Z*-score < −0.93 was determined as the best cut-off for predicting low BMD. In newly diagnosed pediatric IBD, a substantial number of children are likely to have low BMD in prior to initial treatment while lower BMI, longer duration of clinical manifestation, and higher selenium concentration could affect initial BMD status. Routine bone health surveillance from initial IBD diagnosis throughout the treatment’s completion is recommended for preventing the early development of secondary osteoporosis.

## 1. Introduction

Inflammatory bowel disease (IBD) is a chronic inflammatory disorder of the gastrointestinal (GI) tract that can be classified into Crohn’s disease (CD) and ulcerative colitis (UC). Previously, IBD was common only in Western countries. However, since the 2000s, the incidence and prevalence of IBD have steadily increased in Asia, and the gap has narrowed considerably [1]. Pediatric IBD is no longer rare in South Korea. In the United States and Canada, the incidence of pediatric IBD is approximately 10 cases per 100,000 children [2]. The incidence of pediatric IBD in South Korea has also gradually increased and was reported as 13.33 per 100,000 children in multicenter data published in 2020, with no difference in incidence as compared to that in Western countries [3].

Unlike most other chronic disease that develop in later in life, IBD develops during adolescence and young adulthood; therefore, approximately 25% of patients with IBD are first diagnosed before the age of 20 [4]. As growth and development are not yet complete during childhood and adolescence, the effects of IBD-induced inflammation, malnutrition, and medications used for the treatment of patients are inevitably different [5,6,7]. Many reports have revealed that pediatric IBD has a significant impact on patients’ linear growth, pubertal development, nutritional status, and bone health [8,9,10,11]. Therefore, while treating pediatric IBD, it is crucial to monitor, prevent, and treat these secondary problems along with treating the disease [8,12,13,14].

Bone modeling occurs almost exclusively in children and adolescents. Thus, chronic inflammatory diseases at this age may have different consequences on bone metabolism than those in adulthood, affecting the patient’s bone remodeling, modeling, and linear growth [11]. In addition, in healthy children, the earliest ages of bone mineral accrual is 11–14 years for girls and 13–17 years for boys [15]. Considering that the average age at which pediatric IBD is diagnosed to be 12 years, pediatric IBD has a significant impact on bone health [16]. Patients with IBD are more prone to osteopenia and osteoporosis than the general population, especially children with IBD who have significant bone mass deficiency even at diagnosis [17,18,19,20].

The importance of skeletal health in the treatment of pediatric IBD has been increasingly emphasized. Dual-energy X-ray absorptiometry (DXA) is strongly recommended when IBD is first diagnosed and when there are risk factors such as growth failure, amenorrhea, delayed puberty, and a severe inflammatory disease course. In addition to screening at initial diagnosis, obtaining bone mineral content and areal bone mineral density (BMD) of spine or total body less head via DXA scans is recommended every 1–2 years in children and adolescents with IBD if the age- and sex-matched standard deviation (*Z*) score is less than −1.0 at any monitoring period [11,13]. However, the clinical consequences and long-term outcomes remain unknown, and studies related to pediatric IBD and skeletal health are lacking [21,22]. Moreover, most of the research has been conducted in Western countries. Considering that skeletal health has greatly influenced not only by the disease itself, but also on genetics, culture, the environment, including food, and differences in medical facilities, research targeting Asian patients is also necessary. According to Yin et al., the risk factors for low BMD in adult patients newly diagnosed with IBD are menopause, malnourishment, and corticosteroid treatment [23]. To our knowledge, there are very few studies on the skeletal health of pediatric patients with IBD in Asian countries, and no studies have examined the risk factors for BMD in pediatric patients with IBD in South Korea [24,25]. In this study, we evaluated the skeletal health of patients first diagnosed with pediatric IBD in South Korea to identify the prevalence and risk factors for low BMD.

## 2. Materials and Methods

### 2.1. Subjects

The medical records of 51 children and adolescents aged 10–18 years diagnosed with either CD or UC at a single tertiary care center were retrospectively and cross-sectionally reviewed. CD or UC was diagnosed according to the Porto criteria, based on the patient’s clinical, endoscopic, histopathological, and radiographic findings [26]. BMD was measured using dual-energy X-ray absorptiometry (DXA) at the time of IBD diagnosis. We included participants who were newly diagnosed with IBD, and those who were previously diagnosed with systemic conditions that could affect bone health, such as primary bone disorders, leukemia, systemic lupus erythematosus, or nephrotic syndrome, and had undergone therapeutic interventions were excluded. This study was approved by the Institutional Review Board of the Catholic University of Korea (KC23RISI0606) and was conducted in accordance with the principles of the Declaration of Helsinki. 

### 2.2. Data Collection

#### 2.2.1. General Characteristics and Anthropometric Measurements

Baseline clinical data, including age, sex, family history, clinical manifestations, and IBD-associated characteristics, such as colon involvement, luminal behavior, presence of perianal disease, and severity of scoring, were collected. Additionally, data of extra-GI clinical manifestations including oral ulceration, clubbing, erythema nodosum, rash, uveitis, jaundice, hepatomegaly, arthritis, and primary sclerosing cholangitis were collected. Height was measured in centimeters (cm) using a Harpenden Stadiometer (Holtain^®^, Crymych, UK). Weight was measured in kilograms (kg) using a simple weighing scale (CAS^®^, Seoul, Republic of Korea). Body mass index (BMI) (kg/m^2^) was calculated and converted to age- and sex-matched *Z*-scores according to the national growth chart [27].

#### 2.2.2. Bone Assessment

Lumbar spine bone mineral density (LSBMD) and femoral neck bone mineral density (FBMD) were measured in the anterior–posterior direction using DXA (Horizon W DXA system^®^, Hologic Corp., Marlborough, MA, USA) on the same day of endoscopic confirmation of IBD. A single radiographer, blinded to the clinical history of the patients, was responsible for all the BMD measurements. Areal BMD (g/cm^2^) values were converted to age- and sex-matched *Z*-scores based on the national reference data [28]. BMD *Z*-scores were subcategorized as *Z*-scores less than 0, −1.0, and −2.0, whereas low BMD was defined as LSBMD and FBMD *Z*-scores < −1.0. 

#### 2.2.3. Laboratory Assessment

Data on baseline laboratory profiles, including plasma complete blood count; glucose, albumin, total bilirubin, alanine aminotransferase (ALT), aspartate aminotransferase, calcium, phosphorus, bone-specific alkaline phosphatase, C-reactive protein, calcidiol, parathyroid hormone, cyanocobalamin, ferritin, zinc, and selenium levels; and erythrocyte sedimentation rate were collected during the fasting state. Serological markers such as prealbumin, presepsin, procalcitonin, antineutrophil cytoplasmic antibody (ANCA), anti-Saccharomyces cerevisiae antibody (ASCA), and stool calprotectin levels were analyzed. Disease activity was calculated using the Pediatric Crohn’s Disease Activity Index (PCDAI) and Pediatric Ulcerative Colitis Activity Index (PUCAI) scores. Mild, moderate, and severe activity were indicated by PCDAI scores < 30, 30–44, and >45 for CD, and <35, 35–64, and >65 for UC, respectively [26].

### 2.3. Statistics

For all descriptive variables, the normality of the distribution was determined using the Shapiro–Wilk test. The general characteristics of the CD and UC groups were compared using the *t*-test and Mann–Whitney U test for parametric and non-parametric variables, respectively. Subsequently, all IBD subjects underwent the following analyses. The Pearson’s correlation coefficient (*r*) was used to explain the correlation of *Z*-scores of LSBMD and FMBD with clinical and laboratory parameters of patients with IBD, whereas the Chi-squared (χ^2^) test was used to check the correlation of LSBMD *Z*-score of less than 0, −1.0, and −2.0 with categorical variables. Uni- and multivariate regression analyses were performed to estimate the beta coefficients (β) for factors associated with the *Z*-scores of LSBMD and FBMD. Finally, the power of the association between BMI and BMD was determined using odds ratios (OR) and multiple logistic regression analysis. Along with binomial logistic regression analysis, receiver operating characteristic (ROC) curve and area under the curve (AUC) were used to evaluate the optimal cutoff for BMI at IBD diagnosis for predicting low BMD. All statistical analyses were performed using SPSS software (version 24.0; IBM Corp., Armonk, NY, USA).

## 3. Results

### 3.1. Demographic and Clinical Characteristics

Among a total 51 children and adolescents with IBD, baseline characteristics were compared between the CD and UC groups (Table 1). The patients’ sex, age, and family history at the time of diagnosis of IBD did not differ between the CD and UC groups. The frequency of GI-associated manifestations, including abdominal pain, diarrhea, and weight loss, did not differ between the CD and UC groups, except for the number of patients with bloody stools, which was higher in the UC group. In terms of extra-GI-associated symptoms, one patient had erythema nodosum and another patient had arthritis, both of whom had CD. More than half of the patients with CD had both large and small bowel involvement, whereas UC was confined to the large bowel. Most patients with CD showed neither stricturing nor penetrating luminal behavior, and perianal disease was uncommon. Both the PCDAI and PUCAI scores were highest in the category of moderate disease activity.

### 3.2. BMD Status at IBD Diagnosis

**Figure 1:**
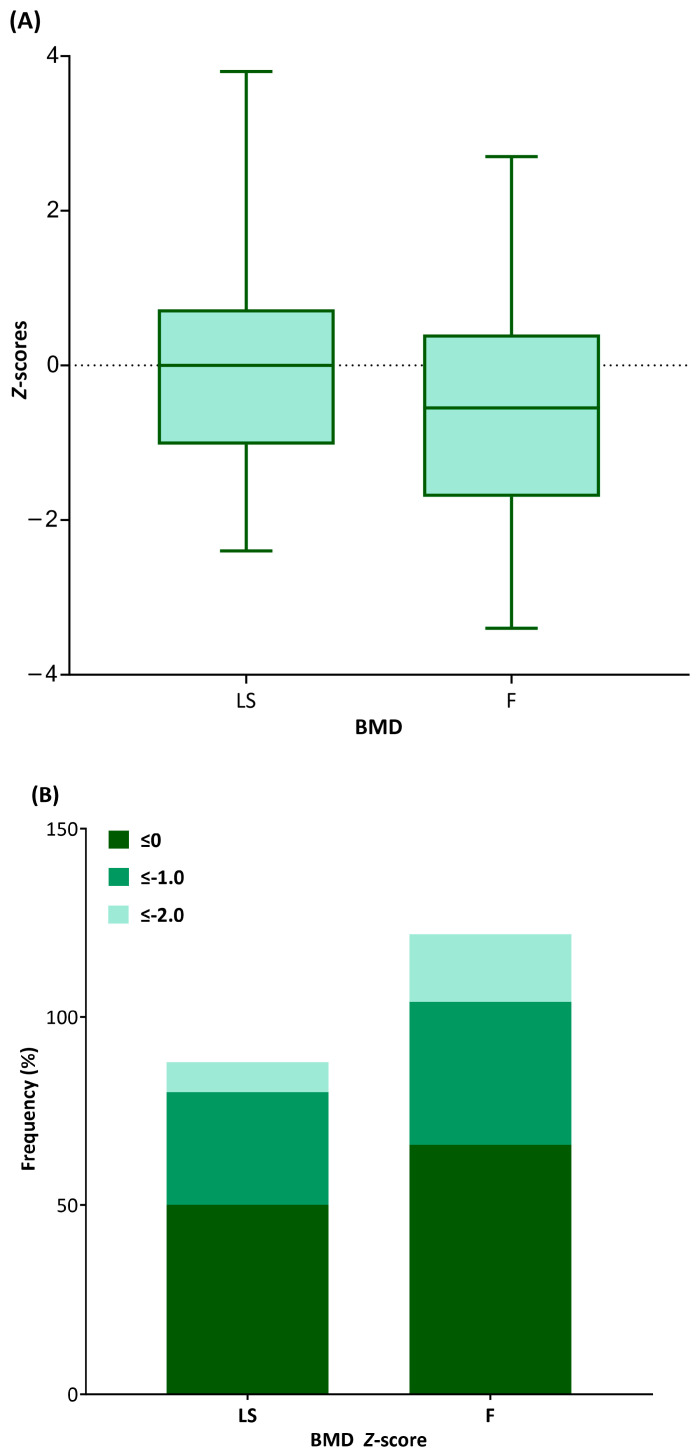
(**A**) Box-and-whisker plots describe bone mineral density (BMD) *Z*-scores for lumbar spine (LS) and femur (F). The lines inside the boxes indicate the mean values, whereas the whiskers indicate the lowest and highest observations. (**B**) The stacked bars represent the frequencies of *Z*-scores less than 0, −1.0, and −2.0 for LSBMD and FBMD in the study population.

**Figure 2:**
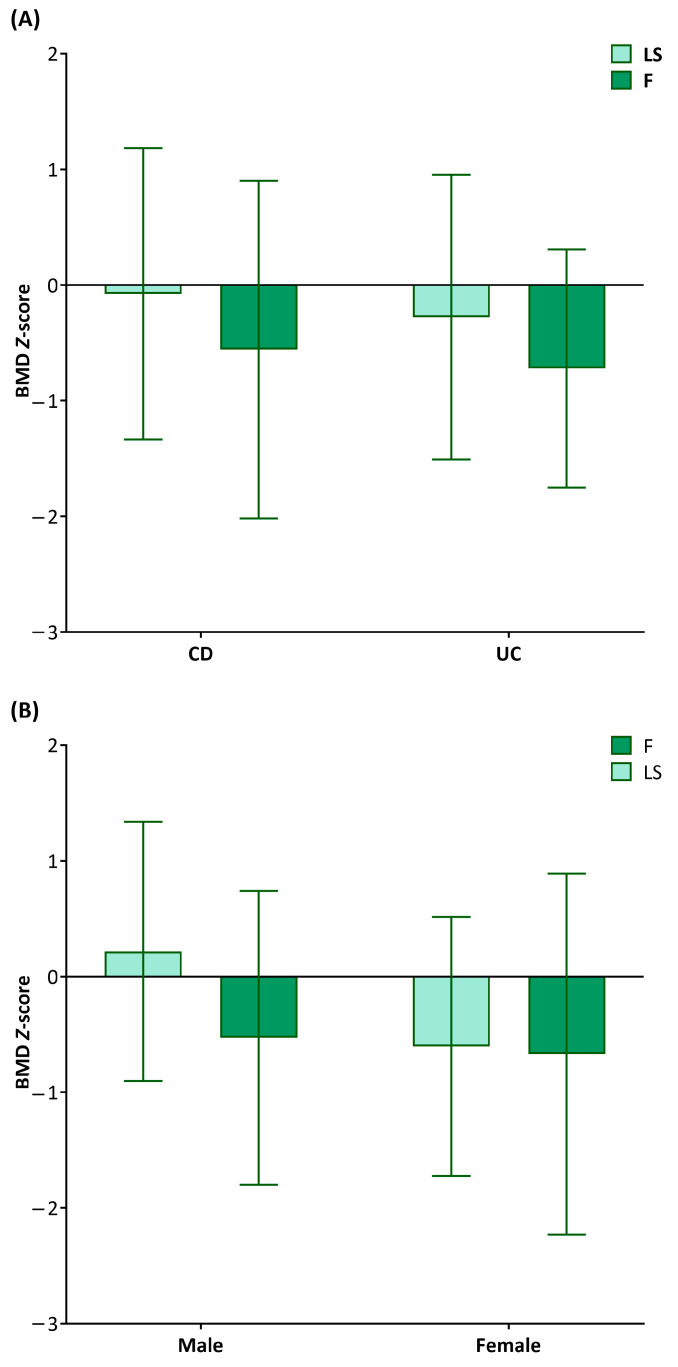
Box-and-whisker plots are drawn to compare bone mineral density (BMD) *Z*-scores for lumbar spine (LS) and femur (F) according to (**A**) disease category and (**B**) gender, where each whisker indicates standard deviation.

The mean *Z*-scores of LSBMD and FBMD were −0.11 ± 1.24 and −0.58 ± 1.38, respectively. More than half of the entire population had scores between 0 and −1.0 (Figure 1). Only 7.8% and 18% of the entire IBD population had *Z*-scores of <−2.0 for the lumbar spine and femur, respectively. The *Z*-scores for both LSBMD and FBMD did not differ between CD and UC groups (−0.08 ± 1.26 vs. −0.28 ± 1.23, *p* = 0.663 for LSBMD *Z*-scores; −0.56 ± 1.46 vs. −0.72 ± 1.03, *p* = 0.751 for FBMD *Z*-scores) (Figure 2A). The LSBMD *Z*-scores in female patients were lower than those in male participants (−0.61 ± 1.28 vs. −0.22 ± 1.12, *p* = 0.02), whereas the FBMD *Z*-scores showed no significant gender difference (−0.67 ± 1.568 vs. −0.53 ± 1.27, *p* = 0.736) (Figure 2B).

### 3.3. Clinical Factors Affecting BMD 

Among the clinical parameters, the BMI *Z*-scores were positively correlated with both LSBMD (*r* = 0.652, *p* < 0.001) and FBMD (*r* = 0.646, *p* < 0.001) *Z*-scores, whereas the duration of clinical manifestations was negatively correlated with the LSBMD (*r* = −0.483, *p* < 0.001) and FBMD (*r* = −0.367, *p* = 0.009) *Z*-scores (Table 2). Among the laboratory parameters, serum ALT and selenium levels were positively correlated with the LSBMD (*r* = 0.315, *p* = 0.026; *r* = 0.477, *p* < 0.001) and FBMD (*r* = 0.34, *p* = 0.016; *r* = 0.375, *p* = 0.009) *Z*-scores. In addition, serum glucose (*r* = 0.283, *p* = 0.047) and albumin (*r* = 0.328, *p* = 0.02) levels were positively correlated with the FBMD *Z*-scores only. No other laboratory parameters showed direct correlations.

The LSBMD and FBMD *Z*-scores were subcategorized into three groups based on their degree, and a contingency table was drawn to determine their correlation with categorical parameters (Table 3). Initial presentation of weight loss at IBD diagnosis was correlated with LSBMD and FBMD *Z*-scores less than 0 (*χ*^2^ = 9.14, *p* = 0.011; *χ*^2^ = 22.9, *p* < 0.001) and −1.0 (*χ*^2^ = 8.98, *p* = 0.011; *χ*^2^ = 11.3, *p* = 0.03), but not with less than −2.0. The presence of extra-GI manifestation was correlated with LSBMD *Z*-scores less than −1.0 (*χ*^2^ = 4.89, *p* = 0.027) and −2.0 (*χ*^2^ = 4.99, *p* = 0.025) and FBMD *Z*-score less than −2.0 (*χ*^2^ = 9.49, *p* = 0.002), respectively. In addition, luminal behavior in CD patients (*χ*^2^ = 5.06, *p* = 0.025), disease activity indices (*χ*^2^ = 7.86, *p* = 0.02) were correlated with FBMD *Z*-score less than −1.0 while bloody stool (*χ*^2^ = 4.7, *p* = 0.03), ASCA (*χ*^2^ = 3.93, *p* = 0.047), and ANCA (*χ*^2^ = 5.09, *p* = 0.024) were correlated with FMBD *Z*-score less than −2.0. Otherwise, no significant correlation between categorical factors and BMD *Z*-score was seen.

In the univariate regression analysis, the height (β = 0.45, *p* < 0.001 for LSBMD and β = 0.38, *p* = 0.013 for FBMD), weight (β = 0.59, *p* < 0.001 for LSBMD and β = 0.61, *p* < 0.001 for FBMD), BMI (β = 0.56, *p* < 0.001 for LSBMD and β = 0.62, *p* < 0.001 for FBMD), serum ALT levels (β = 0.03, *p* = 0.026 for LSBMD and β = 0.04, *p* = 0.016 for FBMD), and selenium levels (β = 0.01, *p* < 0.001 for LSBMD and β = 0.01, *p* = 0.009 for FBMD) were positively associated, whereas the duration of clinical manifestation (β = −1.05, *p* < 0.001 for LSBMD and β = −0.89, *p* = 0.009 for FBMD) was negatively associated with LSBMD and FBMD *Z*-scores (Table 4). In the subsequent multivariate regression analyses, the BMI *Z*-score was an independent factor positively associated with LSBMD and FBMD *Z*-scores (β = 0.39, *p* < 0.001 for LSBMD and β = 0.45, *p* < 0.001 for FBMD). In contrast, the duration of clinical manifestations (β = −0.56, *p* = 0.024 for LSBMD and β = −0.09, *p* = 0.763 for FBMD) and serum selenium level (β = 0.01, *p* = 0.003 for LSBMD and β = 0.01, *p* = 0.051 for FBMD) were other factors that showed significant associations with LSBMD *Z*-scores.

### 3.4. BMI as a Low BMD Indicator

A final logistic regression analysis revealed that BMI is a potential risk factor of LSBMD and FBMD *Z*-scores < −1.0 (Table 5). The unadjusted ORs of BMI < −1 standard deviation (SD) for LSBMD and FBMD *Z*-scores < −1.0 were 11.79 (95% CI = 1.39–99.69, *p* = 0.024) and 4.39 (95% CI = 1.06–18.19, *p* = 0.041), whereas those of <−2 SD were 21.33 (95% CI = 4.32–105.43, *p* < 0.001) and 19.94 (95% CI = 3.65–108.89, *p* < 0.001), respectively. After adjusting for GI-associated and extra-GI manifestation at initial presentation and the duration of clinical manifestation, the OR of BMI < −2.0 SD became significantly greater (OR = 31.97, 95% CI = 3.34–306.46, *p* = 0.003 for LSBMD and OR = 41.45, 95% CI = 2.37–725.9, *p* = 0.011 for FBMD).

**Figure 3:**
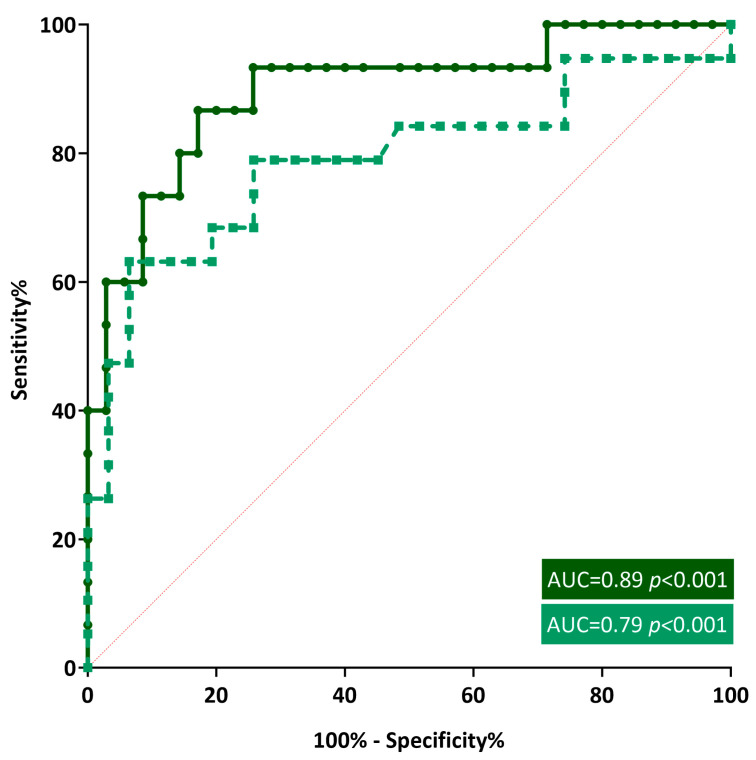
Receiver operating characteristic curves of body mass index for identifying childhood inflammatory bowel disease in patients with low bone mineral density.

According to the ROC curve, the AUCs of BMI were 0.89 (95% CI = 0.79–0.99, *p* < 0.001) and 0.79 (95% CI = 0.65–0.94, *p* < 0.001) for LSBMD and FMBD, respectively (Figure 3). A BMI *Z*-score of less than −0.93 was determined to be the best cut-off for predicting low BMD. The corresponding sensitivities and specificities for each BMI *Z*-score cutoff were 93.33% and 74.29% for LSBMD and 78.95% and 74.19% for FBMD, respectively.

## 4. Discussion

More than one-third of the IBD population was osteopenic, whereas approximately 7.8% was suspected to be severely osteoporotic before proceeding to systemic high-dose glucocorticoid (GC) administration as an initial treatment for IBD. Additionally, we suggest early clinical indicators of low BMD when IBD is newly diagnosed in pediatric populations. Depending on the severity of IBD, it could take years of biological therapy until chronic inflammation is controlled and clinical remission is maintained; thus, close monitoring of either disease- or treatment-associated outcomes becomes critical. As skeletal health can also be threatened, routine bone health surveillance from the beginning of IBD diagnosis throughout the entire treatment course is recommended for the early detection and prevention of osteoporosis.

Along with other chronic conditions, such as neuromuscular, connective tissue, hemato-oncologic, and renal disorders, chronic inflammation characterized by IBD from the beginning of diagnosis is a strong risk factor for secondary osteoporosis. Intestinal homeostasis is regulated by epithelial barrier function, host defense pathways, immune regulation, and tissue repair, and its breakdown caused by deranged cytokine networks leads to IBD [29]. Pro-inflammatory cytokines, including interleukins, tumor necrosis factors, transforming growth factor-alpha, epidermal growth factor, and prostaglandin E2, are key mediators of IBD and are produced by immune responses mediated by T lymphocytes and macrophages. Interleukin-6 levels correlated with sex steroid deficiency, and the relationship between the clinical progression of IBD and the severity of low BMD might be affected by genetic alterations of interleukin-6 and interleukin-1 receptor antagonist genes [30]. In addition to GC’s osteotoxic effects through reduced bone formation and enhanced bone resorption, older age, female sex, smoking, and family history of fractures were independent contributors to low BMD in adult patients with IBD [31]. In both primary and secondary IBD, patients with either a non-traumatic vertebral fracture or a BMD *Z*-score less than −2.0 are indicated for bisphosphonate treatment to prevent the progression of osteoporosis [32].

The most reliable early indicator of low BMD in patients with IBD identified in our study was low BMI, and low BMI *Z*-scores at the time of IBD diagnosis were associated with low BMD. Our findings are consistent with those of previous studies in Western populations and Asian adults [22,24,33]. However, a low BMI at IBD diagnosis could also be an early indicator of low BMD in pediatric patients with IBD in Asia. The optimal cut-off value of the BMI *Z*-score predicting low BMD was confirmed to be −0.93, indicating that the active surveillance of skeletal health and continuous management should proceed, since those with a BMI *Z*-score less than −0.93 were anticipated to have non-traumatic fractures.

The time required for IBD diagnosis varies significantly among patients. In some cases, the symptoms of IBD are very severe from the beginning, and the diagnosis is relatively quick; however, in other cases, the symptoms are not severe, and the diagnosis is made only several years after the onset of symptoms [34]. In addition, each country’s medical environment and economic status may affect the timing of IBD diagnosis. Our results suggest that the longer the duration of clinical manifestations before IBD diagnosis, the more often individuals had a low BMD. This has not been observed in previous skeletal health studies targeting patients diagnosed with IBD for the first time. Early endoscopic diagnosis based on clinical manifestations suggestive of IBD is critical for preventing further skeletal complications.

Selenium is a trace mineral and an indispensable component of various enzymes and proteins [35]. Selenium deficiency is uncommon in the general population but may occur in patients with IBD or chronic kidney disease [36]. Selenium plays an important role in skeletal development, and its deficiency can cause bone damage [37]. Likewise, our findings were consistent in that the selenium level at the time of IBD diagnosis was associated with both LSBMD and FBMD, whereas its deficiency could lead to low BMD. The selenium level in patients with IBD was lower than that in the control group; thus, it may be suggested as a risk factor for osteoporosis, although its relationship with low BMD at the time of IBD diagnosis in the pediatric population has never been elucidated [38,39]. Additional research is needed to determine the role of selenium as an independent indicator to identify the high risk of low BMD when IBD is newly diagnosed in children and adolescents.

Various factors, including chronic inflammation, osteotoxic medication, poor nutrition, and reduced physical activity, contribute to the higher incidence of low BMD in patients with IBD than that in the general population [40,41,42]. Identifying the risk factors associated with low BMD at IBD diagnosis in the pediatric population may be helpful for better understanding the associated pathophysiology and planning subsequent treatment. The bone health of pediatric patients with IBD is increasingly drawing attention in relation to their long-term prognosis, and all children diagnosed with IBD are recommended to undergo either plain radiography of the central bones or DXA screening [11]. However, for various reasons, it is often difficult to measure BMD in all patients in actual practice. Investigating early predictors of low BMD and high-risk groups with low BMD can help create more detailed treatment policies related to the skeletal health of pediatric patients with IBD.

Our study had several limitations. First, this was a retrospective cross-sectional study with a small sample size. Second, levels of physical activity, dietary calcium and vitamin D intakes, and sunlight exposure, all of which are critical factors affecting BMD, were not assessed. Lastly, the assessment of skeletal health was solely based on BMD results acquired using DXA and not on the radiographic findings of vertebral or non-vertebral fractures. Despite this fact, this is the first study to investigate the BMD status in newly diagnosed IBD in the Asian pediatric population. The clinical indicators identified in this study, including low BMI, may provide the opportunity to closely monitor skeletal health before aggressive treatment begins.

## 5. Conclusions

In those with newly diagnosed pediatric IBD, a substantial number of patients are likely to have low BMD before proceeding to remission induction therapy. Among the associated factors, BMI, duration of clinical manifestations, and selenium concentration could affect the initial BMD status, and BMI was considered the strongest indicator of decreased BMD. For patients with a low BMI, routine bone health surveillance from the initial IBD diagnosis to treatment completion is recommended to prevent the early development of secondary osteoporosis.

## Figures and Tables

**Table 1:** Clinical characteristics of the study subjects.

	Total (*n* = 51)		
	CD (*n* = 42)	UC (*n* = 9)	*p*
Male, *n* (%)	26 (61.9)	4 (44.4)	0.346
Age, *y*	13.77 ± 2.35	14.45 ± 2.46	0.437
Family history of IBD, *n* (%)	6 (14.3)	1 (11.1)	0.82
Anthropometry, *Z*-score			
Height	0.32 ± 0.49	0.05 ± 1.25	0.557
Weight	−0.229 ± 1.42	−1.03 ± 1.62	0.266
Body mass index	−0.54 ± 1.41	−1.28 ± 1.54	0.169
Clinical manifestation			
Gastrointestinal-associated, *n* (%)			
Abdominal pain	33 (78.6)	8 (88.9)	0.495
Diarrhea	27 (64.3)	8 (88.9)	0.157
Bloody stool	7 (16.7)	8 (88.9)	<0.001
Weight loss			0.697
None	10 (23.8)	3 (33.3)	
≤10%	16 (38.1)	6 (66.7)	
>10%	15 (35.7)	6 (66.7)	
* Extra-GI-associated, *n* (%)	2 (3.9)	0 (0)	0.532
Duration, *y*	0.58 ± 0.58	0.58 ± 0.57	0.708
Involvement, *n* (%)			<0.001
Colon	2 (4.8)	9 (100)	
Colon and ileum	13 (30.9)	0 (0)	
Colon, ileum, and duodenum or jejunum	27 (64.3)	0 (0)	
^†^ Luminal behavior, *n* (%)	^†^ L		
None	39 (92.9)		
Stricturing	3 (7.1)		
Penetrating	0 (0)		
^†^ Perianal disease, *n* (%)	^†^ *p*		
Yes	16 (38.1)		
No	26 (61.9)		
^‡^ Severity of scoring	^‡^	^‡^	0.792
Mild	13 (30.9)	1 (11.1)	
Moderate	15 (35.7)	6 (66.7)	
Severe	14 (33.3)	2 (22.2)	

^†^ For Crohn disease only. ^‡^ Scoring generated by Pediatric Crohn Disease Activity Index (PCDAI) for Crohn’s disease group and Pediatric Ulcerative Colitis Activity Index (PUCAI) for ulcerative colitis group. * Extragastrointestinal manifestations included oral ulceration, clubbing, erythema nodosum, rash, uveitis, jaundice, hepatomegaly, arthritis, and primary sclerosing cholangitis. All values are expressed as mean ± standard deviation, unless otherwise mentioned. CD, Crohn’s disease; IBD, inflammatory bowel disease; UC, ulcerative colitis.

**Table 2:** Correlation of clinical parameters with bone mineral density *Z*-scores.

	BMD *Z*-Scores			
	LS		RF	
	*r*	*p*	*r*	*p*
Age	0.046	0.752	0.109	0.451
Height	0.463	<0.001	0.350	0.013
Weight	0.704	<0.001	0.649	<0.001
BMI	0.652	<0.001	0.646	<0.001
Duration of CM	−0.483	<0.001	−0.367	0.009
WBC	−0.049	0.736	−0.055	0.704
Hb	0.242	0.091	0.239	0.095
Plt	−0.039	0.79	−0.166	0.248
Glucose	0.178	0.215	0.283	0.047
Albumin	0.229	0.11	0.328	0.02
TB	0.199	0.165	0.111	0.443
AST	0.238	0.096	0.274	0.054
ALT	0.315	0.026	0.34	0.016
Calcium	0.166	0.249	0.214	0.136
Phosphorus	0.162	0.261	−0.008	0.956
BS-ALP	−0.068	0.764	−0.174	0.438
ESR	−0.173	0.229	−0.244	0.088
CRP	−0.07	0.628	−0.105	0.47
Calcidiol	0.046	0.753	−0.008	0.956
Parathyroid hormone	−0.256	0.165	−0.015	0.937
Cyanocobalamin	−0.048	0.745	0.091	0.536
Ferritin	0.038	0.791	−0.022	0.878
Prealbumin	0.099	0.5	0.15	0.305
Zinc	0.143	0.327	0.26	0.156
Selenium	0.477	<0.001	0.375	0.009
Presepsin	−0.047	0.762	0.107	0.488
Procalcitonin	−0.098	0.503	−0.103	0.48
Calprotectin	−0.038	0.791	−0.151	0.295

ALT: alanine transaminase; AST: aspartate transaminase; BMD: bone mineral density; BMI: body mass index; BS-ALP: bone-specific alkaline phosphatase; CM: clinical manifestation; CRP: C-reactive protein; ESR: erythrocyte sedimentation rate; Hb: hemoglobin; LS: lumbar spine; Plt: platelet; RF: right femur; TB: total bilirubin; WBC: white blood cells.

**Table 3:** Contingency table for categorical factors associated with decreased lumbar spine and femoral neck bone mineral density *Z*-scores.

	BMD *Z*-Scores											
	LS						RF					
	≤0		≤−1.0		≤−2.0		≤0		≤−1.0		≤−2.0	
	*χ* ^2^	*p*	*χ* ^2^	*p*	*χ* ^2^	*p*	*χ* ^2^	*p*	*χ* ^2^	*p*	*χ* ^2^	*p*
Family history of IBD	0.76	0.384	0.04	0.849	0.59	0.441	0.91	0.339	0.06	0.802	0.01	0.928
Abdominal pain	2	0.157	0.59	0.44	2.45	0.118	1.43	0.232	4.16	0.041	0.54	0.462
Diarrhea	0.37	0.544	1.42	0.234	0.09	0.754	0.13	0.72	1.69	0.194	0.48	0.487
Bloody stool	0.09	0.758	0.11	0.736	0.83	0.363	1.53	0.216	1.17	0.28	4.7	0.03
Weight loss	9.1	0.011	8.98	0.011	1.57	0.455	22.9	<0.001	11.3	0.003	4.17	0.125
Extragastrointestinal manifestation	2.08	0.149	4.86	0.027	4.99	0.025	1.07	0.3	3.4	0.065	9.49	0.002
Involvement	0.13	0.938	0.49	0.783	1.92	0.383	0.57	0.751	0.21	0.901	2.78	0.249
^†^ Luminal behavior	0.41	0.52	2.19	0.139	3.23	0.072	1.68	0.195	5.06	0.025	0.39	0.53
^†^ Perianal disease	0.27	0.606	1.4	0.236	1.04	0.308	0.13	0.717	2.17	0.141	0.82	0.365
^‡^ Scoring	3.62	0.164	4.86	0.088	2.62	0.27	4.64	0.098	7.86	0.02	1.7	0.427
ASCA	0.29	0.588	3.47	0.063	0.03	0.869	1.12	0.289	1.04	0.308	3.93	0.047
ANCA	1.63	0.201	1.17	0.28	0.69	0.404	0.25	0.617	2.23	0.136	5.09	0.024

^†^ For Crohn’s disease only. ^‡^ Scoring generated by Pediatric Crohn Disease Activity Index (PCDAI) for Crohn’s disease group and Pediatric Ulcerative Colitis Activity Index (PUCAI) for ulcerative colitis group. All values are expressed as mean ± standard deviation, unless mentioned. ANCA, anti-neutrophil cytoplasmic antibody; ASCA, anti-Saccharomyces cerevisiae antibody; IBD, inflammatory bowel disease.

**Table 4:** Univariate and multivariate regression analyses of factors associated with lumbar spine and femoral bone mineral density.

	Univariate				Multivariate			
	*Z*-Scores							
	LSBMD		FBMD		LSBMD		FBMD	
	β (95% CI)	*p*	β (95% CI)	*p*	β (95% CI)	*p*	β (95% CI)	*p*
Height	0.45 (0.2–0.7)	<0.001	0.38 (0.08–0.67)	0.013				
Weight	0.59 (0.42–0.76)	<0.001	0.61 (0.39–0.81)	<0.001				
BMI	0.56 (0.37–0.75)	<0.001	0.62 (0.4–0.83)	<0.001	0.39 (0.19–0.58)	<0.001	0.45 (0.19–0.69)	<0.001
Duration of CM	−1.05 (−1.61–−0.5)	<0.001	−0.89 (−1.54–−0.24)	0.009	−0.56 (−1.09–−0.08)	0.024	−0.09 (−0.7–0.52)	0.763
Glucose	0.01 (0–0.03)	0.215	0.02 (0–0.04)	0.047			0.02 (0–0.04)	0.014
Albumin	0.43 (−0.1–0.98)	0.11	0.69 (0.12–1.28)	0.02			0.26 (−0.31–0.82)	0.361
ALT	0.03 (0–0.06)	0.026	0.04 (0–0.06)	0.016	0.01 (−0.01–0.03)	0.411	0.01 (−0.02–0.04)	0.414
Selenium	0.01 (0–0.03)	<0.001	0.01 (0–0.03)	0.009	0.01 (0–0.02)	0.003	0.01 (0–0.02)	0.051

ALT, alanine transaminase; BMI, body mass index; CM, clinical manifestation; FBMD, femoral bone mineral density; LSBMD, lumbar spine bone mineral density.

**Table 5:** Logistic regression analyses of body mass index when using decreased lumbar spine and femoral bone mineral density *Z*-scores < −1.0 as dependent variables.

	BMD *Z*-Score ≤ −1.0					
	LS			F		
	OR (95% CI)	SE	*p*	OR (95% CI)	SE	*p*
BMI						
Unadjusted						
^†^ <−1 SD	11.79 (1.39–99.69)	1.09	0.024	4.39 (1.06–18.19)	0.73	0.041
^‡^ <−2 SD	21.33 (4.32–105.43)	0.82	<0.001	19.94 (3.65–108.89)	0.87	<0.001
* Adjusted						
<−1 SD	7.93 (0.57–110.68)	1.34	0.124	1.33 (0.19–9)	0.98	0.769
<−2 SD	31.97 (3.34–306.46)	1.15	0.003	41.45 (2.37–725.9)	1.46	0.011

^†^—1 standard deviation equals to body mass index *Z*-score of −0.468. ^‡^—2 standard deviation equals to body mass index *Z*-score of −1.645. * Adjusted for gastrointestinal and extragastrointestinal manifestations at initial presentation and the duration of clinical manifestations. BMD, bone mineral density; BMI, body mass index; CI, confidence interval; F, femur; LS, lumbar spine; SD, standard deviation; SE, standard error.

## Data Availability

The data that support the findings of this study are available from the corresponding author upon reasonable request.
